# Effect of obesity on ability to lower exposure for detection of low‐attenuation liver lesions

**DOI:** 10.1002/acm2.13149

**Published:** 2020-12-28

**Authors:** Brian R. Herts, Andrew Schreiner, Frank Dong, Andrew Primak, Jennifer Bullen, Wadih Karim, Douglas Nachand, Sara Hunter, Mark E. Baker

**Affiliations:** ^1^ Cleveland Clinic Imaging Institute ‐ Desk L10 Cleveland OH USA; ^2^ Department of Medical Physics – Desk AC‐211 Cleveland Clinic Imaging Institute Beachwood OH USA; ^3^ c/o Imaging Institute – Desk AC‐221 Siemens Healthineers Beachwood OH USA; ^4^ Department of Quantitative Health Sciences – JJN3 Cleveland Clinic Cleveland OH USA

**Keywords:** computed tomography, iterative reconstruction, low dose

## Abstract

**Purpose:**

The purpose of this study was to assess the effect of obesity and iterative reconstruction on the ability to reduce exposure by studying the accuracy for detection of low‐contrast low‐attenuation (LCLA) liver lesions on computed tomography (CT) using a phantom model.

**Methods:**

A phantom with four unique LCLA liver lesions (5‐ to 15‐mm spheres, –24 to –6 HU relative to 90‐HU background) was scanned without (“thin” phantom) and with (“obese” phantom) a 5‐cm thick fat‐attenuation ring at 150 mAs (thin phantom) and 450 mAs (obese phantom) standard exposures and at 33% and 67% exposure reductions. Images were reconstructed using standard filtered back projection (FBP) and with iterative reconstruction (Adaptive Model‐Based Iterative Reconstruction strength 3, ADMIRE). A noninferiority analysis of lesion detection was performed.

**Results:**

Mean area under the curve (AUC) values for lesion detection were significantly higher for the thin phantom than for the obese phantom regardless of exposure level (*P* < 0.05) for both FBP and ADMIRE. At 33% exposure reduction, AUC was noninferior for both FBP and ADMIRE strength 3 (*P* < 0.0001). At 67% exposure reduction, AUC remained noninferior for the thin phantom (*P* < 0.0035), but was no longer noninferior for the obese phantom (*P* ≥ 0.7353). There were no statistically significant differences in AUC between FBP and ADMIRE at any exposure level for either phantom.

**Conclusions:**

Accuracy for lesion detection was not only significantly lower in the obese phantom at all relative exposures, but detection accuracy decreased sooner while reducing the exposure in the obese phantom. There was no significant difference in lesion detection between FBP and ADMIRE at equivalent exposure levels for either phantom.

## 1 INTRODUCTION

Recent estimates suggest that obesity will affect more than 40% of the US population by 2030, and medical costs related to obesity in the United States were estimated at more than $140 billion in 2008.^1^, ^2^ Obesity presents problems for all medical imaging modalities but is especially problematic for computed tomography (CT), as the radiation exposure must be increased in obese patients to maintain diagnostic efficacy.^3^ However, concerns about radiation dose may prevent radiologists from increasing exposure to the extent needed.

Iterative reconstruction techniques have been introduced as one potential method for improving contrast‐to‐noise ratio while reducing radiation exposure in CT. However, for the assessment of CT performance with iterative reconstruction at reduced exposure, task‐based studies of image quality are preferred to studies measuring contrast‐to‐noise ratio and subjective assessments of image quality,^4^ as noise equivalence and subjective imaging preference are not sufficient substitutes for diagnostic efficacy. Some examples of task‐based studies assessing CT performance at reduced exposure include those assessing the detection of renal calculi,^5^, ^6^, ^7^ lung nodules,^8^, ^9^ and simulated low‐contrast low‐attenuation (LCLA) liver lesions.^10^, ^11^

Although the goal of modifying protocols to reduce patient exposure in CT is laudable, data are lacking regarding the effect of radiation exposure on overall diagnostic efficacy and the ability to lower radiation exposure in obese patients while maintaining diagnostic efficacy. The purpose of this study was to assess the effect of obesity and iterative reconstruction on the ability to reduce exposure by studying the accuracy for detection of LCLA liver lesions on CT using a phantom model.

## 2 MATERIALS AND METHODS

### 2.A Phantom design

**Fig. 1:**
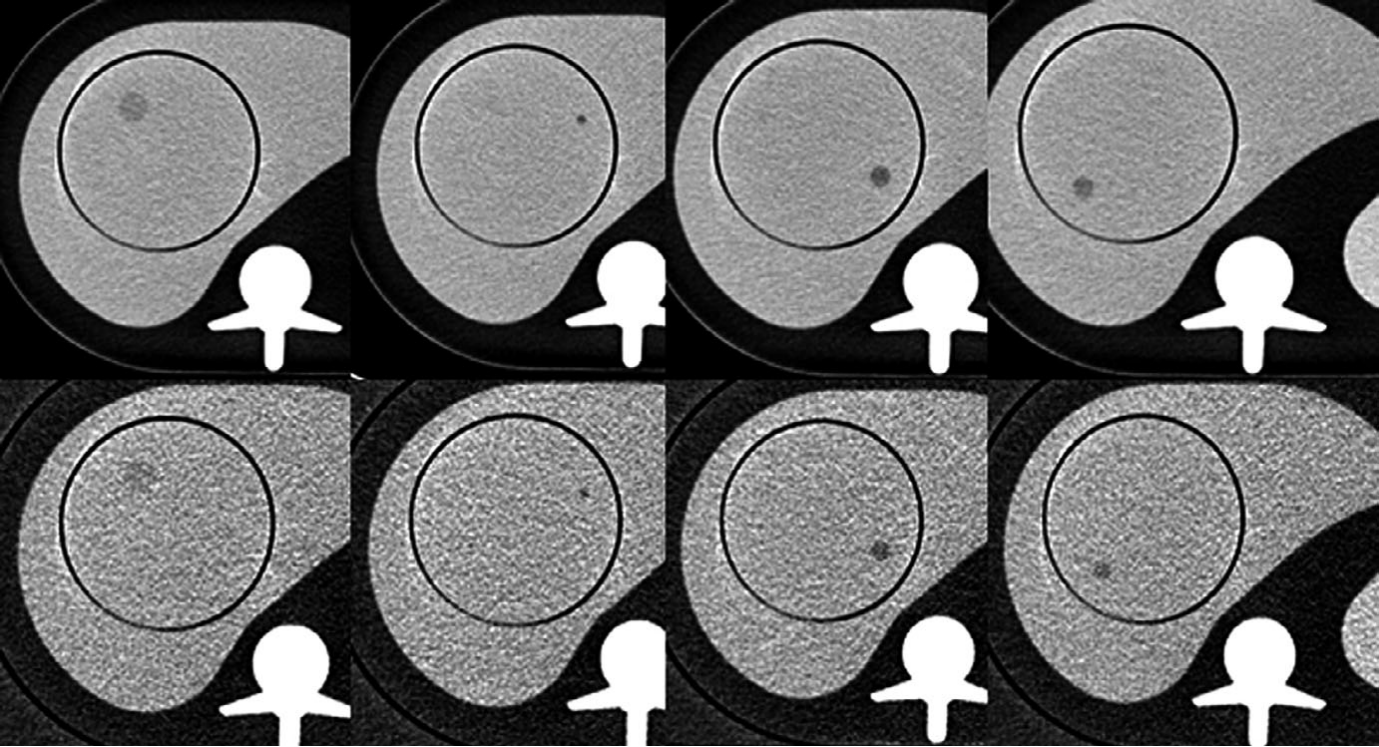
Composite of cross‐sectional images of the liver phantom showing the four different lesions studied, without (upper row) and with (lower row) the fat‐attenuation ring, scanned at maximum dose on the computed tomography scanner (150 kVp at 1725 mAs) to select the center slice for interpretation, and reconstructed with filtered back projection.

An anthropomorphic abdomen phantom (QRM GmbH, Moehrenorf, Germany) was modified to allow a 10‐cm diameter cylindrical insert to be placed in the phantom and rotated; three inserts were fabricated, each containing different combinations of four unique spherical liver lesions (Fig. 1). This design was used to minimize memory bias for lesion location by rotating the insert for different scans and to allow the creation of same‐location lesion present/lesion absent pairs for a concurrent Channelized Hotelling Model Observer (nonhuman observer) study. The inserts were designed in quadrants, with lesions placed in different locations in each quadrant. In each insert, one to three quadrants contained one lesion (but not more than one) and one quadrant was always left blank.

Four LCLA lesions were studied based on the results of a previous published study^10^: 15 mm diameter at 84 HU (–6 HU from the 90‐HU liver background), 10 mm diameter at 78 HU (–12 HU), 10 mm diameter at 72 HU (–18 HU), and 5 mm diameter at 66 HU (–24 HU).

### 2.B Scan technique and reconstruction

All scans were performed in helical mode on a Siemens Somatom Force Scanner (Siemens Healthineers, Forchheim, Germany) at 120 kVp without the use of automated exposure control because the phantom axial image attenuation profile is effectively uniform along the z direction; this also eliminated automated exposure control as a confounding factor.

**Fig. 2:**
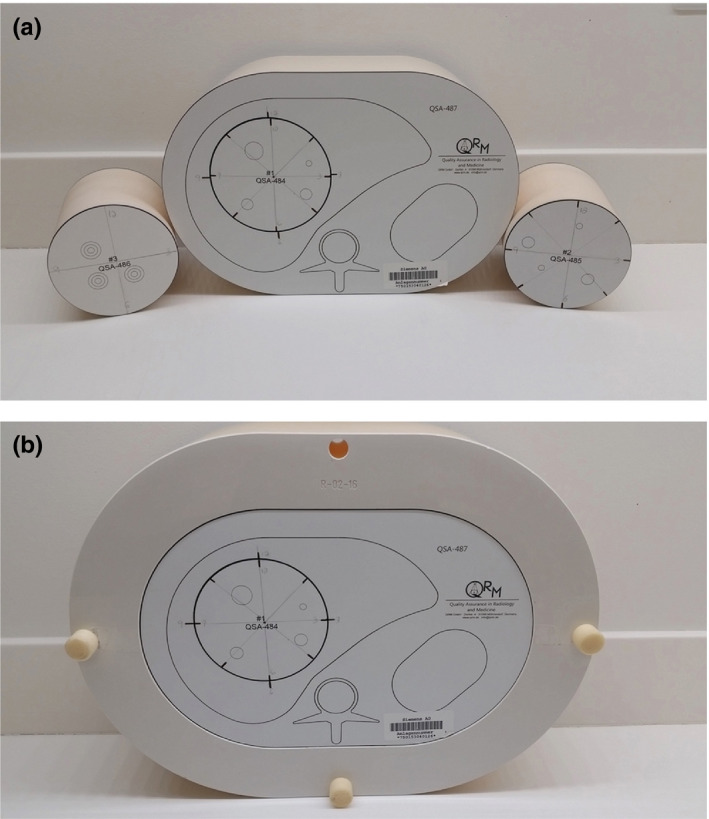
Photographs of the thin (a) and obese (b) phantoms along with additional inserts in (a); the obese phantom includes a 5‐cm thin ring made with material mimicking fat attenuation (–100 HU).

The “thin” phantom was the standard anthropomorphic abdomen phantom (QRM) [Fig. 2(a)]; this oval phantom is 30 cm wide by 20 cm in the anteroposterior dimension and 10 cm in length. Scans of this phantom were performed at 150 mAs (effective mAs, considered standard exposure for the thin phantom for this study), 100 mAs (33% exposure reduction), and 50 mAs (67% exposure reduction). The CTDI_vol_ values for the three exposure levels were 9.9, 6.6, and 3.3 mGy × cm^2^, respectively.

The “obese” phantom was the standard phantom with the addition of a 5‐cm thick ring of fat‐attenuation material on the outside [Fig. 2(b)], resulting in a phantom that was 40 cm wide by 30 cm in the anteroposterior dimension. Scans were performed at 450 mAs (effective mAs, considered standard exposure for the obese phantom for this study), 300 mAs (33% exposure reduction), and 150 mAs (67% exposure reduction). The CTDI_vol_ values for the three exposure levels were 30, 20, and 10 mGy × cm^2^, respectively. These exposures, equal to 3 times the exposures used for the thin phantom, were considered clinically acceptable; to achieve a calculated noise in the center of the obese phantom that was equivalent to that achieved in the thin phantom would have required an approximately 7.4‐fold increase in the exposure to >1100 mAs for full exposure, an exposure level that is not routinely used in or acceptable for clinical practice.

Each scan was performed a minimum of six times at each dose while the lesion insert was rotated among four 90‐degree positions, moving the lesions within the phantom to minimize reader memory bias. The number of scans was determined by our statisticians to allow statistical significance at 10% accuracy loss for a noninferiority study using six readers.^10^ Each scan was reconstructed with 3‐mm slice thickness at 3‐mm intervals, similar to abdominal CT scans performed at our institution, using both a B31f kernel for filtered back projection (FBP) and without and with iterative reconstruction (Adaptive Model‐based Iterative Reconstruction (strength 3, ADMIRE, Siemens Healthineers). Scans were also reconstructed in both the craniocaudal and caudal–cranial directions to provide additional image datasets with variations of the noise pattern in the reconstructed images.

### 2.C Interpretation of images

Six readers who were blinded to the study design interpreted a total of 630 images consisting of a combination of FBP and ADMIRE images from the thin and obese phantoms with different rotations of the inserts. Images were reviewed on PACS workstations approved for clinical use, with the ambient environment set per reader preference. Window width and level settings were preset with our standard liver settings of width of 200 and level of 115, but could be adjusted freely by each reader. The confidence of each reader for determining the presence of a lesion in each of the four quadrants was recorded on a 5‐point Likert scale (0 = lesion definitely absent to 4 = lesion definitely present) using custom Oracle‐based software designed for the project. The readers consisted of two senior radiologists (>25 yr of abdominal imaging experience), two junior radiologists (<10 yr of abdominal imaging experience), and two resident trainees.

### 2.D Prevalence of lesions in the phantom

The prevalence of lesions in the number of total quadrants was chosen to keep the number of interpreted images below 800 (including analysis for the three dose exposures and two reconstruction algorithms). With a lesion prevalence of approximately 40%, the number of images needed per exposure and algorithm was determined to be 105; with the six variables, the total number of images evaluated by each reader was therefore 630. Combinations of 1, 2, and 3 lesions in each image resulted in an overall lesion prevalence of approximately 45% in the thin phantom and 42% in the obese phantom (Table 1).

**Table 1:** Prevalence of lesions in the reader data set^a^ by size and density (90‐HU background).

Lesion diameter and attenuation	Thin phantom	Obese phantom
	Number of lesions (% of quadrants)	Number of lesions (% of quadrants)
15 mm at 84 HU	26 (9.7%)	14 (9.2%)
10 mm at 78 HU	38 (14.2%)	20 (13.2%)
10 mm at 72 HU	30 (11.2%)	16 (10.5%)
5 mm at 66 HU	26 (9.7%)	14 (9.2%)
Empty quadrants (no lesions)	148 (55.2%)	88 (57.9%)
Total number of images (quadrants)	67 (268)	38 (152)
Total lesions (lesion prevalence)	120 (44.8%)	64 (42.1%)

^a^ This 105‐image data set was replicated for two different reconstruction algorithms and three different radiation exposures for a total of 630 images interpreted by each of the six readers.

### 2.E Statistical analysis

A noninferiority model using Holm’s step‐down procedure was created to assess the accuracy of lesion detection with reduced dose for all lesions combined in both the thin and obese phantoms with FBP and ADMIRE. An accuracy loss of <10% was considered noninferior for this study. The Obuchowski–Rockette method for multireader multicase studies was used to compare the readers’ mean receiver operating characteristic (ROC) areas.

## 3 RESULTS

### 3.A Assessment of noninferiority with exposure reduction

For the thin phantom, detection for all four lesions combined was significantly noninferior at 33% and 67% exposure reduction compared to full exposure (*P* < 0.004) for both FBP and ADMIRE (Table 2).

**Table 2:** Noninferiority of reduced‐dose images in the thin phantom for all lesions combined.

Comparison in thin phantom	Difference in mean ROC areas	95% confidence interval for difference	*P* value for noninferiority*, **
FBP			
FBP full exposure vs FBP 33% reduction	0.022	(0.00, 0.04)	<0.0001
FBP full exposure vs FBP 67% reduction	0.073	(0.05, 0.09)	0.0035
FBP full exposure vs ADMIRE 33% reduction	0.011	(–0.01, 0.03)	<0.0001
FBP full exposure vs ADMIRE 67% reduction	0.050	(0.03, 0.07)	<0.0001
ADMIRE			
ADMIRE full exposure vs ADMIRE 33% reduction	0.008	(–0.01, 0.03)	<0.0001
ADMIRE full exposure vs ADMIRE 67% reduction	0.047	(0.03, 0.07)	<0.0001

ADMIRE, adaptive model‐based iterative reconstruction; FBP, filtered back projection; ROC, receiver operating characteristic.

* *P* values adjusted for multiple comparisons using Holm’s step‐down procedure.

** All values were significantly noninferior (*P* < 0.05) for a 10% reduction in ROC area.

For the obese phantom, detection for all four lesions combined was significantly noninferior for a 10% loss of accuracy at 33% exposure reduction compared to full exposure (*P* < 0.0001) for both FBP and ADMIRE but was no longer noninferior at 66% exposure reduction for FBP (0.916 vs 0.779; *P* = 0.9755) and ADMIRE (0.914 vs 0.827; *P* = 0.7353) (Table 3).

**Table 3:** Noninferiority of reduced‐dose images in obese phantom for all lesions combined.

Comparison in obese phantom	Difference in mean ROC areas	95% confidence interval for difference	*P* value for noninferiority*
FBP			
FBP full exposure vs FBP 33% reduction	0.006	(–0.03, 0.04)	<0.0001
FBP full exposure vs FBP 67% reduction	0.137	(0.10, 0.17)	0.9755**
FBP full exposure vs ADMIRE 33% reduction	–0.004	(–0.04, 0.03)	<0.0001
FBP full exposure vs ADMIRE 67% reduction	0.089	(0.05, 0.13)	0.7353**
ADMIRE			
ADMIRE full exposure vs ADMIRE 33% reduction	–0.006	(–0.04, 0.03)	<0.0001
ADMIRE full exposure vs ADMIRE 67% reduction	0.087	(0.05, 0.12)	0.7353**

ADMIRE, adaptive model‐based iterative reconstruction; FBP, filtered back projection; ROC, receiver operating characteristic.

* *P* values adjusted for multiple comparisons using Holm’s step‐down procedure.

** Comparisons that were not proven statistically noninferior (effectively inferior) (*P* > 0.05) for a 10% reduction in ROC area.

### 3.B Reader accuracy

**Fig. 3:**
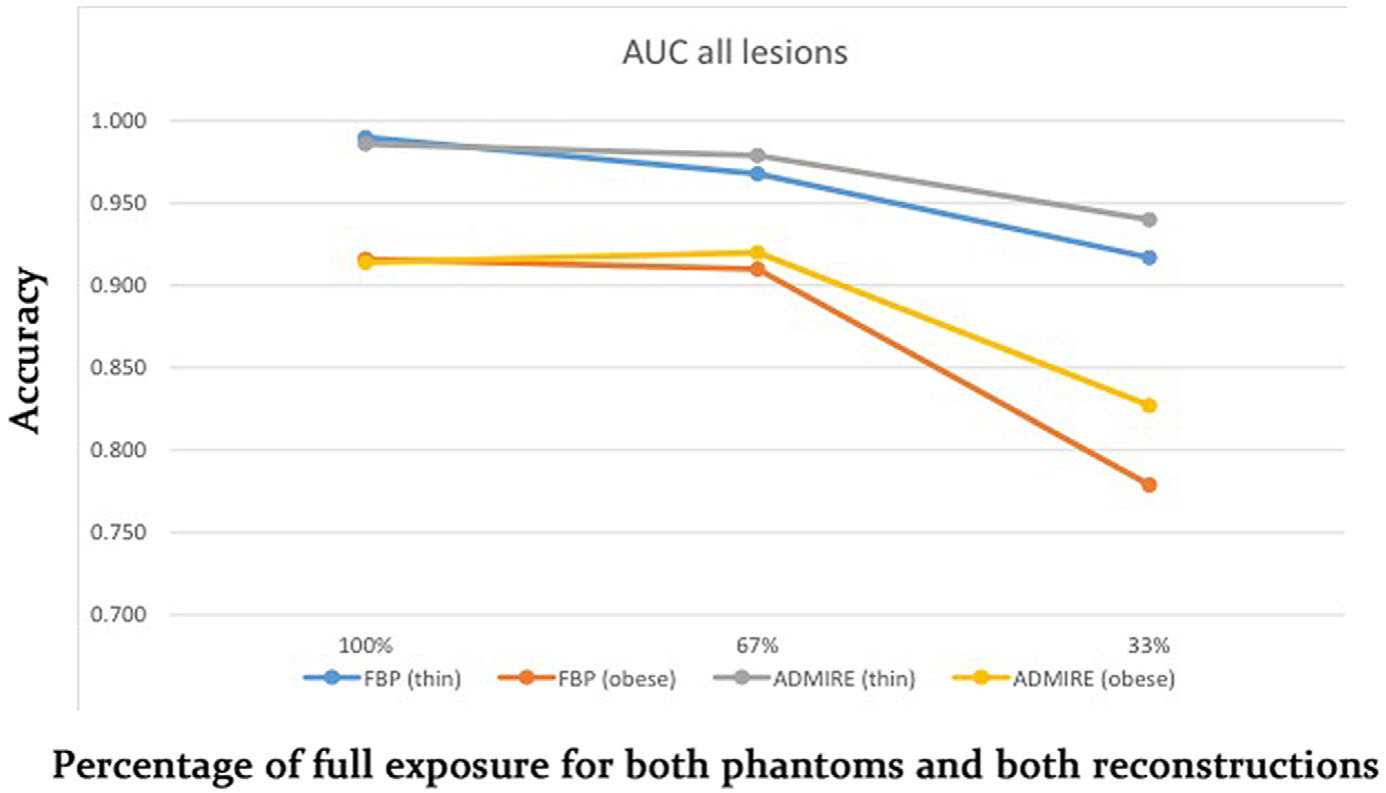
Line chart showing the effect of reduced exposure and iterative reconstruction on reader detection (accuracy) of low‐contrast low‐attenuation liver lesions as exposure decreases. AUC, area under the curve.

With FBP, the mean area under the curve (AUC) was consistently lower for the obese phantom at each exposure level, even with the threefold increased exposure; at “full” exposure, for example, the AUC was 0.990 at 150 mAs for the thin phantom vs 0.916 at 450 mAs for the obese phantom. Similar results were seen with ADMIRE (e.g., mean AUC was 0.986 at 150 mAs for the thin phantom vs 0.914 at 450 mAs for the obese phantom) (Table 4, Fig. 3).

**Table 4:** Direct comparison of accuracy for lesion detection in thin and obese phantoms.

Reconstruction method and relative exposure level	Thin phantom	Obese phantom	Difference (95% confidence interval)*
FBP			
FBP full exposure	0.990	0.916	–0.074 (–0.04, –0.12)
33% exposure reduction	0.968	0.910	–0.058 (–0.04, –0.12)
67% exposure reduction	0.917	0.779	–0.128 (–0.15, –0.47)
ADMIRE			
ADMIRE full exposure	0.986	0.914	–0.074 (–0.03, –0.12)
33% exposure reduction	0.979	0.920	–0.059 (–0.03, –0.11)
67% exposure reduction	0.940	0.827	–0.113 (–0.11, –0.22)

ADMIRE, adaptive model‐based iterative reconstruction; FBP, filtered back projection.

* All comparisons were significantly lower for the obese phantom (*P* < 0.05).

### 3.C Reader confidence

All six readers used the “indeterminate” score (3 on the 5‐point Likert scale) at a higher frequency for the obese phantom than for the thin phantom at full exposure for both FBP (obese phantom, 13%; thin phantom, 8%) and ADMIRE (obese phantom, 14%; thin phantom, 8%).

## 4 DISCUSSION

In this study, the accuracy for detection of LCLA liver lesions simulated in a phantom was shown to decrease and become no longer noninferior with 67% exposure reduction in the obese phantom when it remained noninferior for the thin phantom. This was despite a threefold increase in the base‐line exposure. In other words, exposure reductions in obese patients are more likely to result in lower diagnostic efficacy sooner than in thin patients.

Iterative reconstruction preserved accuracy more at the lower exposures than higher exposures, as expected. While the improvement was not statistically significant, the trend suggests that when reducing exposure with CT, it is prudent to choose iterative reconstruction techniques over FBP for this type of lesion detection task.

Increasing radiation exposure for CT scans is necessary to maintain diagnostic efficacy in obese patients given the physics of CT. This was shown by Zhang et al^12^ for the detection of subcentimeter lesions as the size of a phantom was increased from 25 to 35 cm in diameter. However, radiologists are understandably reluctant to increase exposure to the extent needed to obtain equivalent diagnostic efficacy in obese patients.

Iterative reconstruction techniques have been introduced as a method for reducing radiation exposure while limiting concomitant increases in noise, but these techniques have not been as effective at maintaining diagnostic efficacy as anticipated. In general, iterative reconstruction has at best modestly improved confidence in LCLA liver lesion detection without significantly improving the accuracy of detection.^10^ Although one study showed subjective improvements in image quality with a noise‐reducing iterative reconstruction algorithm,^13^ several studies have shown little improvement in objective, task‐based assessment of image quality with noise‐reducing algorithms.^11^, ^14^, ^15^, ^16^, ^17^, ^18^ One study showed increased detection with iterative reconstruction; however, this finding was for lesions 6 mm and smaller with low‐contrast differentials.^15^ These lesion sizes are not clinically relevant for most uses of abdominal imaging, and the degradation of lesion detection at lowered exposures in obese patients was not evaluated in the study.

In another study, Samei et al^19^ evaluated model‐based iterative reconstruction, adaptive statistical iterative reconstruction, and FBP, showing improved image noise and some task‐based improvement with these techniques, but this study did not evaluate the effect of obesity. Viry et al,^20^ on the other hand, showed that iterative reconstruction could have more of an effect toward maintaining diagnostic efficacy at lower exposure for an obese phantom model than for a standard phantom model.

None of these previous studies directly evaluated how obesity affects the ability to lower exposure and maintain LCLA liver lesion detection, either without or with iterative reconstruction. In this study, the decrease in accuracy (loss of noninferiority) of lesion detection with reduced exposure became significant sooner in the obese phantom than it did in the thin phantom, even at three times the exposure levels. Importantly, this study illustrates that exposure reductions in obese patients are more likely to result in lower diagnostic efficacy than they do in thin patients.

There are several limitations to this study. First and foremost, this is a phantom model of a liver lesion; the noise patterns in phantoms and human subjects are likely less uniform^21^ and therefore the results may be different in human subjects, although likely worse. We also did not increase the exposure for the obese phantom enough to achieve an image noise in the center of the phantom similar to that achieved in the thin phantom, as this would have required exposures markedly higher than are clinically used or acceptable. However, we were studying the relative rate of deterioration of lesion detection with reductions in exposure levels, not the specific exposure levels. Furthermore, maintaining image noise is not an adequate substitute for maintaining diagnostic efficacy. Another limitation is that only subcutaneous obesity was simulated for this study; the fat placed on the outside of the abdominal phantom simulated an increase in subcutaneous fat only. We were unable to study an equivalent diameter increase with fat attenuation within the abdominal cavity because of the phantom's physical design restrictions. Therefore, we do not know whether the findings would differ for predominantly intra‐abdominal obesity, although one previous study demonstrated a theoretical effect of patient fat distribution on x‐ray exposures.^22^ Other limitations include the small number of readers and the use of a static, single image rather than dynamic (scrolling) assessment. However, a previous study demonstrated no significant advantage with scrolling image assessment.^23^ We also evaluated only lesion detection, not lesion characterization, but any degradation in the ability to characterize lesions would likely be greater than the ability to simply detect lesions. Finally, we studied only one CT scanner from one vendor, and thus the findings are not necessarily generalizable to other CT scanners or protocols, as has been shown previously.^12^

## 5 CONCLUSION

The results from this study using an anthropomorphic abdominal phantom show that subcutaneous fat plays an important role in the detection of LCLA liver lesions, requiring a markedly higher radiation exposure to achieve similar but still reduced detection rates at standard clinical exposures. Moreover, for the obese phantom, the accuracy of detection decreased more rapidly as exposure was reduced and thus exposure reduction should be employed cautiously in obese patients. The risks of underdiagnosis in obese patients should be weighed against the risks of radiation exposure when evaluating CT protocols.

## AUTHORS CONTRIBUTIONS

Brian R. Herts: Study design, manuscript writing and final approval, and agrees to be accountable for all aspects of the work in ensuring that questions related to the accuracy or integrity of any part of the work are appropriately investigated and resolved. Andrew Schreiner: Study design, data acquisition, analysis, intellectual content for the manuscript, editing and final approval of the manuscript, and agrees to be accountable for all aspects of the work in ensuring that questions related to the accuracy or integrity of any part of the work are appropriately investigated and resolved. Frank Dong: Study concept and design, analysis and interpretation, intellectual content for the manuscript, editing and final approval of the manuscript, and agrees to be accountable for all aspects of the work in ensuring that questions related to the accuracy or integrity of any part of the work are appropriately investigated and resolved. Andrew Primak: Study concept and design, data acquisition and interpretation, intellectual content for the manuscript, editing and final approval of the manuscript, and agrees to be accountable for all aspects of the work in ensuring that questions related to the accuracy or integrity of any part of the work are appropriately investigated and resolved. Jennifer Bullen: Study design, data analysis and interpretation, editing and final approval of the manuscript, and agrees to be accountable for all aspects of the work in ensuring that questions related to the accuracy or integrity of any part of the work are appropriately investigated and resolved. Wadih Karim, Douglas Nachand, and Sara Hunter: Data acquisition, final approval of the manuscript, and agrees to be accountable for all aspects of the work in ensuring that questions related to the accuracy or integrity of any part of the work are appropriately investigated and resolved. Mark E. Baker: Study concept, manuscript writing and final approval, and agrees to be accountable for all aspects of the work in ensuring that questions related to the accuracy or integrity of any part of the work are appropriately investigated and resolved.

## CONFLICT OF INTEREST

Dr. Brian R. Herts receives grant funding from Siemens Healthineers for research in CT. Dr. Andrew Primak is an employee of Siemens Healthineers.
